# 8-*O*-(*E*-*p*-methoxycinnamoyl)harpagide Inhibits Influenza A Virus Infection by Suppressing Intracellular Calcium

**DOI:** 10.3390/molecules26041029

**Published:** 2021-02-15

**Authors:** Eun-Bin Kwon, Hye-Jin Yang, Young-Soo Kim, Wei Li, Jang-Gi Choi

**Affiliations:** Korean Medicine (KM) Application Center, Korea Institute of Oriental Medicine, Daegu 41062, Korea; wrld2931@kiom.re.kr (E.-B.K.); hjyang@kiom.re.kr (H.-J.Y.); yskim527@kiom.re.kr (Y.-S.K.)

**Keywords:** influenza A virus, 8-*O*-(*E*-*p*-methoxycinnamoyl)harpagide, intracellular calcium, reactive oxygen species, M2 ion channel

## Abstract

Calcium (Ca^2+^) dependent signaling circuit plays a critical role in influenza A virus (IAV) infection. The 8-*O*-(*E*-*p*-methoxycinnamoyl)harpagide (MCH) exhibits pharmacological activities that exert neuroprotective, hepatoprotective, anti-inflammatory and other biological effects. However, not have reports of antiviral effects. To investigate the antiviral activity of MCH on IAV-infected human lung cells mediated by calcium regulation. We examined the inhibitory effect of MCH on IAV infections and measured the level of viral proteins upon MCH treatment using Western blotting. We also performed molecular docking simulation with MCH and IAV M2 protein. Finally, we analyzed MCH’s suppression of intracellular calcium and ROS (reactive oxygen species) in IAV-infected human lung cells using a flow cytometer. The results shown that MCH inhibited the infection of IAV and increased the survival of the infected human lung cells. The levels of IAV protein M1, M2, NS1 and PA were inhibited in MCH-treated human lung cells compared to that in infected and untreated cells. Also, docking simulation suggest that MCH interacted with M2 on its hydrophobic wall (L40 and I42) and polar amino acids (D44 and R45), which formed intermolecular contacts and were a crucial part of the channel gate along with W41. Lastly, MCH inhibited IAV infection by reducing intracellular calcium and mitochondrial Ca^2+^/ROS levels in infected human lung cells. Taken together, these data suggest that MCH inhibits IAV infection and increases the survival of infected human lung cells by suppressing calcium levels. These results indicate that MCH is useful for developing IAV treatments.

## 1. Introduction

Influenza A virus (IAV) is a significant pathogen that infects the respiratory tract and causes influenza, characterized by fever, headache, muscle pain and other conditions. IAV continues to circulate in the human population despite pre-immune responses by vaccines and the resulting annual influenza outbreak is associated with up to 500,000 deaths worldwide [1]. Moreover, these vaccines cannot protect against arising influenza pandemic and currently used antiviral drugs have limitations due to resistance and side effects [1]. Therefore, new options for fighting influenza infection are needed and the success of these efforts will depend on a detailed understanding of virus-host cell interactions.

Calcium (Ca^2+^) is essential for viral entry, viral gene replication, virion maturation and release. Alteration of host cell Ca^2+^ homeostasis is one of the strategies that viruses use to regulate host cell signaling mechanisms. Viruses aid the viral life cycle by intercepting calcium channels or pumps and inhibiting T cell responses, anti-apoptosis and other persistent functions by interfering with host cells’ Ca^2+^ homeostasis [2]. Recent reports have shown that voltage-dependent Ca^2+^ channels are crucial for IAV entry into host cells [3]. IAV infection induces Ca^2+^ influx into cells and intracellular Ca^2+^ levels can modulate intracellular uptake of IAV. Also, chelators such as 1,2-bis(2-aminophenoxy)ethane-N,N,N’,N’-tetraacetic acid (BAPTA-AM), ethylene glycol-bis(β-aminoethyl ether)-N,N,N′,N′-tetraacetic acid (EGTA) and Ca^2+^ channel blocker (CCB) verapamil inhibit IAV infection [4]. Moreover, Ca^2+^ flux from outside to the inside of cells may regulate IAV entry and infection [5]. Therefore, we aimed to find a drug that inhibits IAV infection through calcium regulation using compounds found in natural products.

*Scrophularia buergeriana* (*S. buergerina*) distributed in eastern Asia such as Korea, Japan, Taiwan and China. Roots of *S. buergerina* have been control sore throat and neuralgia, neuroprotection and antioxidation activities [6,7]. The roots of *S. buergerina* have the many active compounds including phenylpropanoids, iridoids and *E*-*p*-methoxycinnamic acid [8]. 8-*O*-(*E*-*p*-methoxycinnamoyl)harpagide (MCH) used in this study is an iridoid glycoside and it has been reported to be isolated from *S. buergerina* roots. In addition, the antioxidant activity and neuroprotective effects have been reported but no mention of antiviral effects [9,10]. In this study, we investigated whether MCH isolated from *S. buergerina* roots inhibits influenza A virus infection through calcium regulation and its associated phenotypes.

## 2. Results

### 2.1. MCH Inhibits IAV Infection in Human Lung Cells

**Figure 1:**
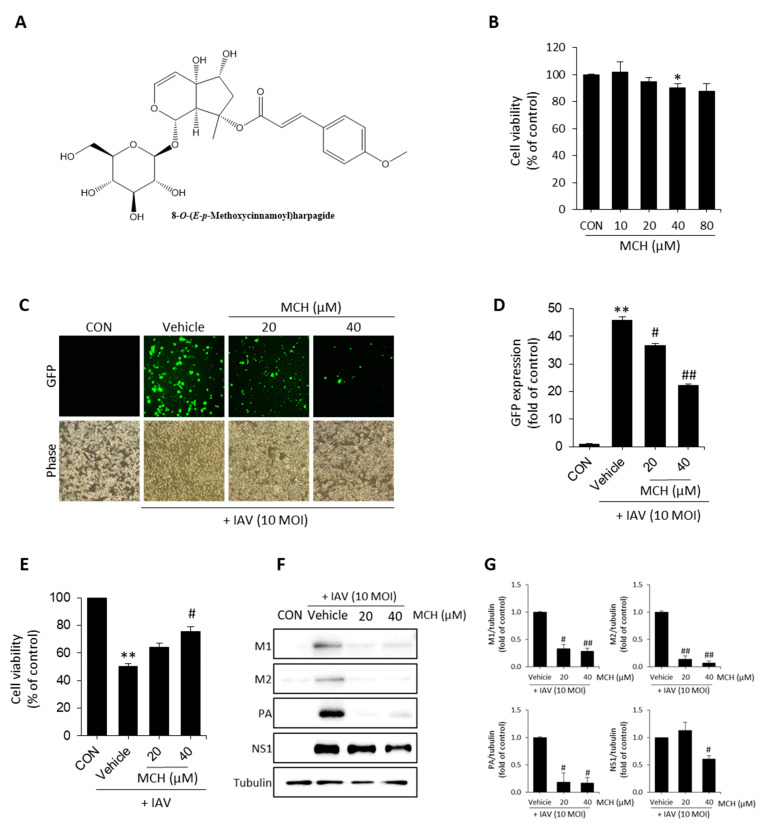
Antiviral effect of 8-*O*-(*E*-*p*-methoxycinnamoyl)harpagide (MCH) on influenza A virus (IAV)-infected A549 cells. (**A**) Structure of MCH. (**B**) Cells were treated with MCH at various concentrations for 24 h. Determination of cytotoxicity using MTT assay. (**C**,**D**) Cells were infected with GFP encoded-H1N1 (A/PR8/34) virus for 2 h and then treated with MCH for 24 h before fluorescence microscopy and quantification using a flow cytometer. (**E**) Determination of antiviral effect. (**F**,**G**) Western blotting of viral expressions and then quantification using the Image J software. The bar graphs show the means ± SD of three independent experiments [** *p* < 0.01 compared with the control (DMSO), # *p* < 0.05 and ## *p* < 0.01 compared with the vehicle (IAV-infected control)].

We confirmed cytotoxicity using 3-(4,5-dimethylthiazol-2-yl)-2,5-diphenyl tetrazolium bromide (MTT) assay before antiviral effect experiments of the MCH isolated from S. buergerina (Figure 1A) in A549 cells. As shown in Figure 1B, MCH exhibits cytotoxicity at 80 μM (approximately 87% versus the control group), so we proceeded with concentrations of 20 μM and 40 μM. In the antiviral experiment, green fluorescent protein (GFP) encoded H1N1 (A/PR8/34) virus was infected for 2 h and then treated with MCH at 20 μM and 40 μM before incubation for 24 h. After incubation, the GFP level was measured using a fluorescence microscope and flow cytometer. The GFP level was decreased by 20.2% and 51.5% after MCH treatment at 20 μM and 40 μM, respectively, compared with vehicle (the virus treatment group) in IAV-infected A549 cells (Figure 1C,D). Also, we were investigated that antiviral effect of MCH on infected with IAV. The results, MCH was shown to restore cell viability in a dose-dependent manner (Figure 1E). We confirmed viral protein expressions such as M1, M2, NS1 and PA using Western blotting to demonstrate the antiviral effect of MCH. As shown in Figure 1F,G, MCH dose-dependently inhibited all expression of viral proteins. Furthermore, MCH significantly inhibited M1 and M2 proteins.

### 2.2. MCH May Inhibit the Function of Ion Channel by Binding onto M2 Protein

**Figure 2:**
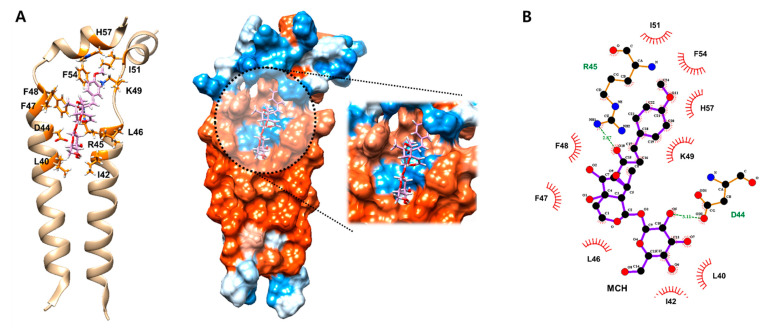
Docking simulation of MCH on the active site of the M2 protein. (**A**,**B**) Computational structure prediction for docking between MCH and the M2 protein of influenza virus H1N1. The binding interaction between them was further investigated with LigPlot+ v1.4.5.

The M2 proton channel of influenza A virus is essential component of viroporin. The function of several viroporin including M2 are known as virus entry, virus morphogenesis and release, regulation of apoptosis and destruction of calcium homeostasis [11]. Based on previous results that MCH significantly inhibited expression of M2 proton protein. Therefore, we investigated the binding mode of MCH to predict its possible mechanisms for inhibiting the function of M2 protein, which is an important ion channel for influenza virus infection, through in silico docking simulation and pharmacophore analysis. MCH potentially interacted with M2 ion channel protein by nine hydrophobic interactions (L40, I42, L46, F47, F48, K49, I51, F54 and H57) and two hydrogen bonds (D44 and R45) (Figure 2A,B). In particular, in silico docking simulation suggest that MCH may inhibit the function of M2 ion channel protein by interacting with its hydrophobic wall (L40 and I42) and polar amino acids (D44 and R45) which form intermolecular contacts and are an crucial part of channel gate along with W41 [12]. These data suggest that MCH might suppress IAV infection by inhibiting M2 protein of IAV in A549 cells.

### 2.3. MCH Decreased Intracellular Calcium and Mitochondrial Stress in IAV-infected A549 Cells

**Figure 3:**
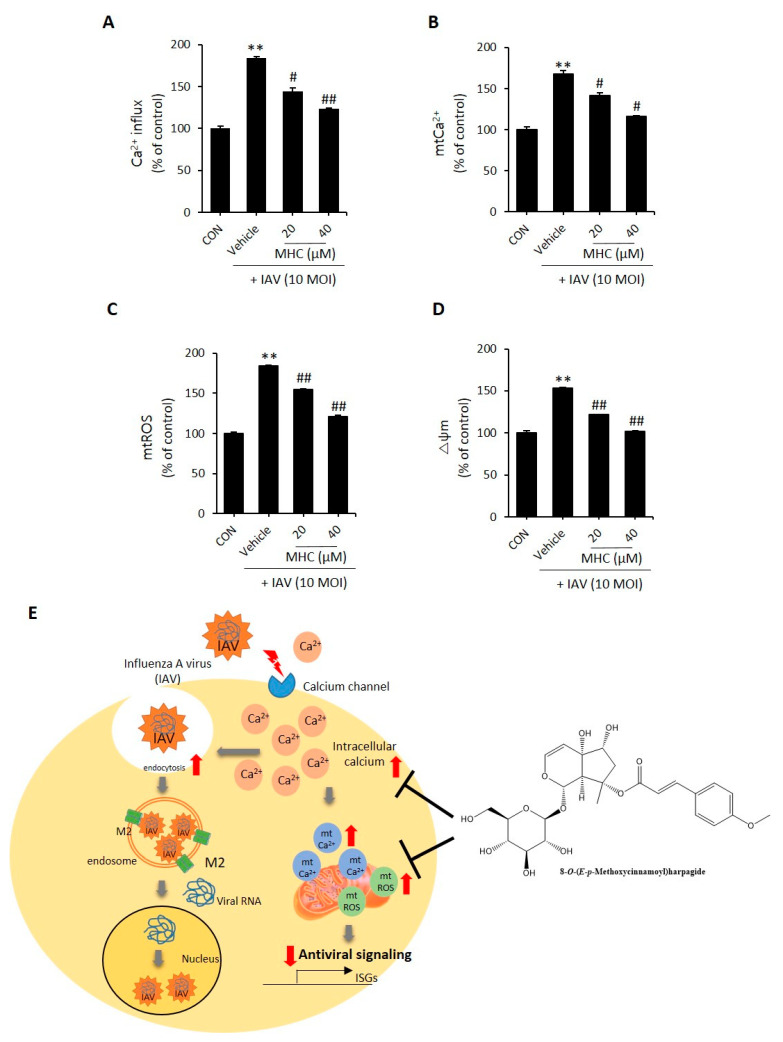
A549 cells. Cells were infected with GFP encoded-H1N1 (A/PR8/34) virus for 2 h and then treated with MCH for 24 h before staining with a fluorescent dye. (**A**) Intracellular Ca^2+^ was measured using Fluo-4AM. (**B**) Mitochondrial Ca^2+^ was measured using Rhod-2 as a selective mtCa^2+^ staining dye. (**C**) Mitochondrial ROS was measured using mitoSOX as a selective mtROS staining dye. (**D**) Mitochondrial membrane potential (MMP, △ψm) was stained with Dioc6(3). (**E**) Graphical abstract of MCH. All data were detected and quantified using a flow cytometer. The bar graphs show the mean ± SD of three independent experiments [** *p* < 0.01 compared with the control (DMSO), # *p* < 0.05 and ## *p* < 0.01 compared with the vehicle (IAV-infected control)]

According to previous reports, IAV infection increases intracellular calcium. Also, chelators such as BAPTA-AM reduced IAV infection in cells [3]. Following previous reports, we determined whether MCH suppressed intracellular calcium in IAV-infected A549 cells. Treatment using MCH for 24 h after IAV infection for 2 h in A549 cells and then intracellular calcium was measured using Fluo-4AM dye. As shown in Figure 3A, MCH dose-dependently reduced intracellular Ca^2+^, which was increased by IAV infection. The excess production of intracellular Ca^2+^ taken in by the mitochondria leads to mitochondrial stress, including mitochondrial calcium (mtCa^2+^) and ROS (mtROS) [13]. Therefore, we evaluated whether intracellular calcium induced by IAV affects mitochondrial calcium and ROS using a fluorescent reagent. As a result, MCH dose-dependently reduced mtCa^2+^ and mtROS compared with the vehicle group (Figure 3B,C). Furthermore, mitochondrial stress interferes with MMP. Therefore, we used the dioc6(3) ((3,3’-Dihexyloxacarbocyanine Iodide) reagent to confirm that MCH restores the MMP that was disrupted due to IAV infection. As shown in Figure 3D, MCH restored the MMP disrupted due to IAV infection in a dose-dependent manner. As shown Figure 3E, intracellular Ca^2+^ increased by the IAV induced the influx of IAV and mitochondrial stress which, aggravates intracellular antiviral signaling. However, MCH can potential inhibits viral infection by reduced intracellular Ca^2+^ and mitochondrial stress. Taken together, these data suggest that MCH inhibits IAV infection by reducing intracellular calcium and mitochondrial Ca^2+^/ROS in IAV-infected A549 cells.

## 3. Discussion

Prevention and treatment efforts against viral infections are challenged by high mutation rates of viral proteins. The host calcium apparatus proteins (calcium-permeable channels or calcium pumps) required for viral replication and release are potential therapeutic targets but have not been changed. For example, verapamil, amlodipine and diltiazem, which are widely used in treating cardiovascular diseases as Ca^2+^ channel blockers, inhibits various viral infections. However, the calcium channel family is continuously being updated with the discovery of new members and the virus has a very complicated influence on the calcium environment in the host cells. Both upstream messengers and downstream Ca^2+^ binding proteins are pharmacological intervention targets [2]. Studies are recently underway on how calcium-mediated viroporin acts in various host signaling pathways such as viral entry, autophagy and inflammasome activation [11]. Recent studies have provided new insights into the role of calcium and viroporin-induced elevation in promoting viral replication and reversing cellular processes that contribute to pathogenesis [11]. Therefore, Ca^2+^ mediated viroporin can be a significant target for antiviral drug development because it performs an essential function in viral replication and entry.

We showed that MCH has a potent antiviral activity in IAV-infected A549 cells. MCH reduces intracellular Ca^2+^, mitochondrial Ca^2+^ and ROS (increased by IAV infection) and restores MMP. Also, we observed that MCH suppressed viral protein and significantly inhibited M2 viroporin. Altogether, results obtained suggest that MCH decreased IAV infection by reducing Ca^2+^ mediated M2 viroporin. However, we only identified phenotypic aspects. Therefore, further study is needed on the antiviral effect though the mechanism and the correlation between calcium and viroporin.

Iridoid derivatives are found in various plants and exist as monoterpenoid types of cyclopentanopyran and glycosides. Iridoids exhibits pharmacological activities, such as neuroprotective effects, hepatoprotective effects, anti-inflammatory activities, hypoglycemic and hypolipidemic activities and antitumor activities [14]. Previous reports have shown that the C10 type iridoid glycosides inhibit IAV and HIV-1 infection [15,16]. MCH, a C9 type iridoid, is structurally similar to the C10 type. The C9 type iridoids have been reported to have neuroprotective effects, insecticidal activity and anti-inflammatory; however, their antiviral effects have not been reported [9,17]. At the time of writing, there have been no report on the antiviral effect of C9 type iridoid glycoside. These results also directly prove that the iridoid derivatives (C9 and C10 type) have a potent antiviral activity

## 4. Materials and Methods

### 4.1. Materials

3-[4,5-Dimethylthiazol-2-yl]-2,5 diphenyl tetrazolium bromide (MTT), dimethyl sulfoxide (DMSO) purchased form Sigma-Aldrich (Saint Louis, MO, USA). Enhanced chemiluminescence (ECL) was obtained from Thermo (Waltham, MA, USA). Flou-4 AM, Rhod-2 and DioC6(3) were purchased from Invitrogen (Carlsbad, CA, USA). Pro-Prep protein extraction solution was purchased from Intron Biotechnology (Seoul, Korea). Antibodies of M1, M2, PA and NS1 was obtained from GeneTex (lrvine, CA, USA). 8-*O*-(*E*-*p*-methoxycinnamoyl)harpagide was isolated from *S. buergerina* roots and identified by an author (Dr. Wei Li).

### 4.2. Cell and Virus

Human lung epithelial cell line A549 was obtained from ATCC (American Type Culture Collection, Manassas, VA, USA). A549 cells RPMI1640 containing 10% FBS (Fetal Bovine Serum, Gibco, Grand Island, NY, USA) and 1% antibiotic-antimyotic at 37 ℃ under 5% CO_2_ atmosphere. Influenza virus strains A/PR/8/34 and A/PR/8/34-green fluorescence protein (GFP, H1N1) were used in previous studies [18,19]. Influenza virus was propagated in the allantoic fluid of 10-day-old chicken embryos [18,19].

### 4.3. Cell Viability and Antiviral Effect

Cells were treated with various concentration (10–80 μg/mL) of MCH or various volumes (0.1–100 μL) of IAV for 24 h before MTT assay when cells were treated with MTT solution (final 0.5 mg/mL) at 37 ℃ for 30 min. After incubation, purple formazan was dissolved in DMSO and absorbance was read at 540 nm using ELISA plate reader (Epoch, Bioteck, Winooski, VT, USA). The antiviral effect was investigated after infected with IAV in the TCID_50_ volume before treatment with MCH at 20 and 40 μM using MTT assay [18].

### 4.4. Flow Cytometry Analysis

Intracellular and mitochondrial calcium were measured using 1 μM Flou-4 AM and 5 μM Rhod-2 at 37 ℃ for 30 min in IAV-infected A549 cells, respectively. Also, mitochondrial ROS and mitochondrial membrane potential (MMP, △ψm) was determined using 1 μM MitoSOX and 40 nM DioC6(3). After incubation, cells were harvested and then washed with DPBS (without Ca^2+^ and Mg^2+^). Fluorescence was detected using a flow cytometer (cytoFLEX, Beckman Coulter Inc., Pasadena, CA, USA).

### 4.5. Immunoblotting

Cells were lysed using Pro-Prep containing protease and phosphatase inhibitor in ice for 30 min before centrifugation (13,200 rpm, for 30 min at 4 °C) and then quantified using the Braford’s method. After SDS-PAGE separation, samples were transferred to polyvinylidene difluoride membranes (PVDF, Millipore, MA, USA), which were then blocked in 0.5 × Ez-Block Chemi (Amherst, NY, USA). Detection was done using ChemiDoc imaging system (UVITEC, Cleaver scientific Ltd., Rugby, UK) with ECL kit after the membrane was incubated with specific primary and secondary antibodies.

### 4.6. In Silico

Docking simulation of MCH on the predefined binding site of M2 ion channel protein was conducted based on its crystal structure (PDB code: 2RLF) [12] using SwissDock [20] and binding mode with the lowest energy score was selected. The binding interaction was further investigated using LigPlot+ v1.4.5 [21] and amino acid residues related to this interaction were indicated with red (hydrophobic interactions) and green (H-bonds).

### 4.7. Statistical Analysis

Data were presented as means ± standard deviation (SD). Graphs were generated using Microsoft Excel (Microsoft Corp., Redmond, WA, USA). Statistical analysis was performed using Student’s *t*-test for the in vitro experiments. Differences were considered significant at *p* < 0.05 (*) and *p* < 0.01 (**).

## 5. Conclusions

Our findings are the first on the antiviral effect of 8-*O*-(*E*-*p*-methoxycinnamoyl)harpagide potentially through Ca^2+^-mediated M2 modulation in IAV-infected A549 cells. This study provides a basis for developing IAV treatments but requires further study.

## Data Availability

No new data were created or analyzed in this study. Data sharing is not applicable to this article.
